# A multilayer approach to multiplexity and link prediction in online geo-social networks

**DOI:** 10.1140/epjds/s13688-016-0087-z

**Published:** 2016-07-26

**Authors:** Desislava Hristova, Anastasios Noulas, Chloë Brown, Mirco Musolesi, Cecilia Mascolo

**Affiliations:** 1 Computer Lab, University of Cambridge, 15 JJ Thompson Ave, Cambridge, CB3 0FD UK; 2 Data Science Institute, University of Lancaster, South Drive, Lancaster, LA1 4YW UK; 3 Department of Geography, University College London, Gower Street, London, WC1E 6BT UK

**Keywords:** online social networks, media multiplexity, multilayer networks, link prediction

## Abstract

Online social systems are multiplex in nature as multiple links may exist between the same two users across different social media. In this work, we study the geo-social properties of multiplex links, spanning more than one social network and apply their structural and interaction features to the problem of link prediction across social networking services. Exploring the intersection of two popular online platforms - Twitter and location-based social network Foursquare - we represent the two together as a composite *multilayer online social network*, where each platform represents a layer in the network. We find that pairs of users connected on both services, have greater neighbourhood similarity and are more similar in terms of their social and spatial properties on both platforms in comparison with pairs who are connected on just one of the social networks. Our evaluation, which aims to shed light on the implications of multiplexity for the link generation process, shows that we can successfully predict links across social networking services. In addition, we also show how combining information from multiple heterogeneous networks in a multilayer configuration can provide new insights into user interactions on online social networks, and can significantly improve link prediction systems with valuable applications to social bootstrapping and friend recommendations.

## Introduction

Online social media has become an ecosystem of overlapping and complementary social networking services, inherently multiplex in nature, as multiple links may exist between the same pair of users [1]. Multiplexity is a well studied property in the social sciences [2] and it has been explored in social networks from Renaissance Florence [3] to the Internet age [4]. Despite the broad contextual differences, multi-channel ties are consistently found to exhibit greater intensity of interactions across different communication channels, which is related to a stronger social bond [2, 5]. In this work, we explore how we can leverage multiplex tie strength through the geographic and social interactions of users and apply it to the classic networks problem of link prediction [6].

Link prediction systems are key components of social networking services due to their practical applicability to friend recommendations and social network bootstrapping, as well as to understanding the link generation process. Link prediction is a well-studied problem, explored in the context of both OSNs and location-based social networks (LBSNs) [6–9]. However, only very few link prediction works tackle multiple networks at a time [10–13], while *most link prediction systems only employ features internal to the network under prediction*, without considering additional link information from other OSNs.

Recently, empirical models of multilayer networks have emerged to address the multi-relational nature of social networks [1, 14]. In such models, interactions are considered as layers in a systemic view of the social network. Despite the observable multilayer nature of online social networks (OSNs) as a system [1, 15, 16], there is little empirical work exploiting data-driven applications in the domain of multilayer OSNs, especially with respect to how location-based and social interactions are coupled in the online social space [10, 17]. Most empirical multilayer social network literature considers multiple dimensions of the same platform [14, 18], whereas we are interested in interactions across different platforms. In the few exceptions where multiple platforms are considered [19], the same properties of social interactions are examined across, whereas our interests lie in using heterogenous interactions from different platforms (both social and geographic) and their multiplex properties.

Media multiplexity [4] is the principle that tie strength is observed to be greater when the number of media channels used to communicate between two people is greater (higher multiplexity). In [2] the authors studied the effects of media use on relationships in an academic organisation and found that those pairs of participants who utilised more types of media (including email and videoconferencing) interacted more frequently and therefore had a closer relationship, such as friendship. More recently, multiplexity has been studied in light of multilayer communication networks, where the intersection of the layers was found to indicate a strong tie, while single-layer links were found to denote a weaker relationship [5]. The strength of social ties is an important consideration in friend recommendations and link prediction [20], and we employ the previously understudied tie multiplexity properties of OSNs to such ends in this work.

In this work, we explore multilayer networks with heterogeneous layers and apply media multiplexity theory to study the social and geographical features of pairs of users and their application to link prediction across online social networks. Unlike previous work [12, 18, 19], we frame the multilayer link prediction problem across online social network platforms and apply media multiplexity as a measure of tie strength, showing its applicability to link prediction in the geo-social domain. We find that pairs of users with links on both Twitter and Foursquare exhibit significantly higher interactions on both social networks than those pairs of users with a link on just one or the other in terms of number of mentions and colocations within the same venues, as well as a lower distance and higher number of common hashtags in their tweets. In our evaluation, we use these interaction features to predict Twitter links from Foursquare features and vice versa, and we achieve this with AUC scores up to 0.86 on the different datasets, which is just as good as predicting links internal to the network on Twitter and almost as good for Foursquare ($\mathrm{AUC}=0.86$ for Twitter and $\mathrm{AUC}=0.88$ for Foursquare). In predicting links which span both networks, we achieve the highest AUC score of 0.88 from our multilayer features set which is higher than results for each single network, suggesting that multilayer frameworks can be a useful tool for social bootstrapping and friend recommendations due their comprehensive perspective on the online social ‘ecosystem’.

## Multilayer online social network

The social network of human interactions is usually represented by a graph $G(V,E)$ where the nodes in set *V* represent people and the edges *E* represent interactions between them. While this representation has been immensely helpful for the uncovering of many social phenomena, it is focused on a single-layer abstraction of human relations. In this section, we describe a model, which represents link multiplexity by supporting multiple friendship and interaction links across heterogenous online social network platforms.

**Figure 1:**
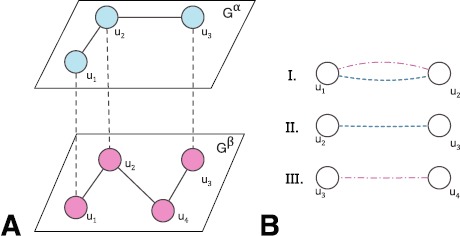
**Multilayer model.** Multilayer model of OSNs (Panel Figure A) with different link types (Panel Figure B): I. Multiplex link; II. Single-layer link on $G^{\alpha}$; and III. Single-layer link on $G^{\beta}$.

We represent the parallel interactions between nodes across OSNs as a *multilayer network* $\mathcal{M}$, or an ensemble of *M* graphs, each corresponding to a distinct layer as ${\mathcal{M}} = \{G^{1},\ldots,G^{\alpha},\ldots,G^{M}\}$. We indicate the *α*-th layer of the multilayer as $G^{\alpha}(V^{\alpha}, E^{\alpha})$, where $V^{\alpha}$ and $E^{\alpha}$ are the sets of vertices and edges of the graph $G^{\alpha}$. Figure 1(A) illustrates the concept by showing how two graphs $G^{\alpha}$ and $G^{\beta}$ are coupled by common neighbours, while some links may be present or absent across the two graphs. As this represents the general case of online social networks, members need not be present at all layers and the multilayer network is not limited to two layers. While each platform can be explored separately as a network in its own right, this does not capture the dimensionality of online social life, which spans across many different platforms.

Figure 1(B) illustrates three link types for the case of a two layer network. Firstly, we define a *multiplex link* between two nodes *i* and *j* as a link that exists between them *at least in two layers* $\alpha, \beta\in\mathcal{M}$. Second, we consider that a *single-layer link* between two nodes *i* and *j* exists if the link appears *only in one layer* in the multilayer social network. In systems with more layers, multiplexity can take on a value depending on how many layers the link is present on [5]. Since our model is applied to online social media, the number of layers can be expected to remain in the single digits due to cognitive limits in human interaction [21]. This will ensure that with each additional layer, the value of link between two individuals increases and information is added to their tie strength [2].

### The multilayer neighbourhood

Following our definition of a multilayer online social network, we can extend the ego network of a node to a *multilayer neighbourhood*. While the simple node neighbourhood is the collection of nodes one hop away from the ego, we define the multilayer *global neighbourhood* (denoted by *GN*) of a node *i* as the total number of unique neighbours across network layers:

1 $$ \Gamma_{GNi} = \bigl\{ j \in V^{\mathcal{M}} : e_{i,j} \in E^{\alpha\cup\beta}\bigr\} , $$

where given layer *α* and layer *β*, we denote the set of all links present in the multilayer network as $E^{\alpha\cup\beta}$. This allows us to reason about the full global connectivity across layers of the system.

We can similarly define the *core neighbourhood* (denoted by *CN*) of a node *i* across layers of the multilayer network as:

2 $$ \Gamma_{CNi} = \bigl\{ j \in V^{\mathcal{M}} : e_{i,j} \in E^{\alpha\cap\beta}\bigr\} , $$

where we define the set of multiplex links as $E^{\alpha\cap\beta}$. Although we weigh all edges equally, we could also take into account the level of multiplexity in geo-social systems with more layers, similarly to [5]. We can further consider the set of all single-layer links on layer *α* only as $E^{\alpha\backslash\beta}$. This simple formulation allows for powerful extensions of existing metrics of neighbourhood similarity. We can consider the Jaccard similarity of two users *i* and *j*’s global neighbourhoods as:

3 $$ \textit{sim}_{GNij} = \frac{|\Gamma_{GNi} \cap\Gamma_{GNj}|}{|\Gamma_{GNi} \cup\Gamma_{GNj}|}, $$

where the number of *common* friends is divided by the number of *total* friends of *i* and *j*. The same can be done for the core degree of two users.

We can further consider the multilayer Adamic/Adar index for link likelihood [22], which takes into account the overlap of two neighbourhoods based on the popularity of common friends (originally through web pages) in a single-layer network as:

4 $$ aa\_{\textit{sim}}_{GNij} = \sum_{z \in\Gamma_{GNi} \cap\Gamma_{GNj}} \frac{1}{\log(|\Gamma_{GNz}|)}, $$

where it is applied to the global common neighbours between two nodes but can be equally applied to their core neighbourhoods. Both the Jaccard similarity and the Adamic/Adar index have been shown to be effective in solving the link prediction problem in both social and location-based networks [6, 9]. In the present work, we aim to show its applicability to the multilayer space in predicting online social links across and between Twitter and Foursquare - two heterogenous social networking platforms.

## Dataset

Twitter and Foursquare are two of the most popular social networks, both with respect to research efforts and user base. They have distinct broadcasting functionalities - microblogging and venue check-ins. While Twitter can reveal a lot about user interests and interactions, Foursquare check-ins provide a proxy for human mobility. In Foursquare users check-in to venues that they visit through their location enabled devices, and share their visit of a place with their connections. Foursquare is two years younger than Twitter and its broadcasting functionality is exclusively for mobile users (50M to date^1^), while also 80% of Twitter’s 284M users are active on mobile.^2^ Twitter generally allows anyone to ‘follow’ and be ‘followed’, where followers and followed do not necessarily know one another. On the other hand, Foursquare supports undirected links, referred to as ‘friendship’ in the service. A similar undirected relationship can be constructed from Twitter, where a link can be considered between two users if they both follow each other reciprocally [23]. Since we are interested in ultimately in predicting friendship, we consider only reciprocal Twitter links throughout this work.

Our dataset was crawled from the public Twitter and Foursquare APIs between May and September 2012 for three major US cities, where tweets and check-ins were downloaded for users who had checked in during that time, and where those check-ins were shared on Twitter. We initially identified Foursquare users on Twitter by hashtags that pertain to the Foursquare service and then continuously crawled their tweets over the four month period. Therefore, our dataset contains a subset of Foursquare users who publicly share their check-ins via the Twitter service, who are estimated to be 20-25% of the Foursquare user base [24]. This allows us to study the intersection of the two networks through users who have accounts and are active on both Twitter and Foursquare. Tweets were divided into *check-ins* and *tweets* depending on whether the content of the tweet was a Foursquare check-in or not. A tweet is in the form (*userId, mentions, hashtags*), where we do not consider the actual content of the tweet but only if it mentions another user or identifies with a topic through Twitter’s hashtag (#) paradigm of topics. Check-ins are in the form (*userId, venueId, coordinates, timestamp*) where we consider the temporal and spatial aspects of the check-in and not its semantic properties. At the end of the period, we also crawled the social network of each user in our dataset on both platforms by obtaining the user ids of their followers and who they are following as well as Foursquare friends of up to one hop in the network. Our dataset does not contain bots or other automated accounts as only real users post content through Foursquare due to its mobile application context.

Table 1 shows the details for each city, in terms of activity and venues, multilayer edges and degrees for each network, where $E^{T \cap F}$ denotes the set of edges, which exist on both Twitter and Foursquare, $E^{T \backslash F}$ and $E^{F \backslash T}$ are the sets of edges on Twitter only and Foursquare only respectively.

**Table 1:** **Dataset properties: number of users (nodes); number of multiplex links (edges); number of Twitter and Foursquare only edges; average global and core degrees; activity and venues per city.**

**Property**	**New York**	**Chicago**	**SF**	**All**
$|V^{\mathcal{M}}|$	6,401	2,883	1,705	10,989
$|E^{T \cap F}|$	9,101	5,486	1,517	16,104
$|E^{T \backslash F}|$	13,623	7,949	1,776	23,348
$|E^{F \backslash T}|$	6,394	4,202	863	11,459
$\langle k_{GN}\rangle$	4.55	6.12	2.44	4.63
$\langle k_{CN}\rangle$	1.42	1.9	0.89	1.47
*tweets*	2,509,802	1,288,865	632,780	4,431,447
*checkins*	228,422	105,250	46,823	380,495
*venues*	24,110	11,773	6,934	42,817

## Properties of multiplex links

Our first goal is to gain insight into the geo-social structural and interaction properties of multiplex links in the multilayer social network and how they differ from other link types. We study the three types of links as described in our multilayer model above: multiplex links across both Twitter and Foursquare, which we denote as *tf* for simplicity; single-layer links on Foursquare only (denoted as *fo*); single-layer links on Twitter only (denoted as *to*), and compare these to unconnected pairs of users (denoted as *na*). We use the insight gained from the discriminative power of each feature to interpret the results of our link prediction tasks defined in the following section.

### Link multiplexity and structural similarity

**Figure 2:**
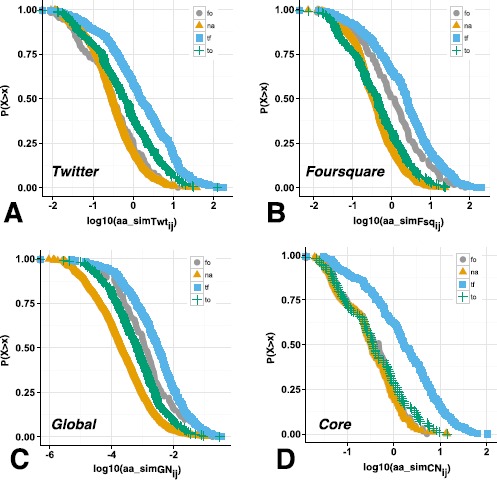
**CCDF of the Adamic/Adar overlap metric.** Complementary cumulative distribution function of the log Adamic/Adar index for the different network configurations, grouped by link type - Twitter overlap (A), Foursquare overlap (B), Global overlap (C), Core overlap (D). Each figure shows the fraction of links with an $aa_{\textit{sim}}$ value greater than *x*.

The number of common friends between two individuals has been shown to be an important indicator of a link in social networks [6]. Moreover, the neighbourhood overlap weighted on the popularity of common links between two users has been shown to be a good predictor of friendship in online networks [22]. Figure 2 shows the cumulative distribution of the Adamic/Adar index of neighbourhood similarity across the various single and multilayer configurations of the networks at hand and each of the four link types. Figure 2(A) and (B) shows the cumulative distribution over the single-layer configurations of Twitter and Foursquare respectively, while Figure 2(C) and (D) shows the distribution over the core and global multilayer configurations. These plots allow us to reason about the fraction of pairs of users with an Adamic/Adar index greater than a certain threshold which relates to the way that features are ranked in a machine learning framework.

Each figure shows the fraction of Adamic/Adar indices greater than the given threshold. In Figure 2(A) we can see that 25% of Twitter user pairs (to) have an overlap of 10^0.3^ or greater, while 25% of multiplex tie pairs (tf) have 10^1^ or higher. Those pairs that are not connected (na) and those which are only connected on Foursquare (fo) have a similarly lower Adamic/Adar threshold of 10^0^. The results over different fractions of user pairs remain consistent where multiplex tie pairs (tf) always have a higher Adamic/Adar index threshold than Twitter only (to), Foursquare only (fo) and no link (na) pairs, based on the CCDF curves. These results are analogous for the Foursquare network, where we have an Adamic/Adar index of approximately 10^1^ for 25% of multiplex pairs, closely followed by Foursquare only (fo) pairs and then Twitter only (to) and na user pairs with a value of 10^0^. From the two single layer configurations, we can see that multiplex links always exhibit the highest structural similarity, followed by links native to the platform and then exogenous and finally unconnected user pairs.

With respect to our multilayer configurations, we can see that user pairs in Figure 2(C), where the global connectivity between the two services is considered, have a similar arrangement in terms of curves as the single-layer configurations. The main differences, however, come from the greater distinction between non-present links (na) and single-layer links (fo and to) than in the single network configurations where exogenous links and non-existent links had similar distribution. In particular, we can see that in Figure 2(C) 50% of user pairs which are not connected have an Adamic/Adar index of 10^−4^ or greater, whereas 50% of single-layer links (fo and to) have 10^−3^ or higher and finally multiplex link pairs (tf) have an index of 10^−3.5^ or greater. On the other hand, in the core configuration in Figure 2(D) we can see a division between multiplex link types and all other link types, where 25% of all pairs of all multiplex ties (tf) have an index of approximately 10^1^ or greater while all other link types have a lower threshold of 10^0^ or higher. While this is somewhat expected, it shows that the core configuration is a good proxy for multiplex ties. In agreement with previous studies of tie strength [20], we observe that multiplex links share greater structural similarity than other link types across network configurations and this will be s useful property in our link prediction problem.

### Link multiplexity and interaction

The volume of interactions between users is often used as a measure of tie strength [25]. In this section we compare how the volume of geo-social interactions on Twitter and Foursquare discriminate between the presence of the various link types. We extract a number of interaction features from the two services, which we will examine in the following section in light of their predictive power in addition to the structural features analysed above. These interaction features are:

- *Number of mentions:* The number of instances in our dataset in which user *i* has mentioned user *j* on Twitter during the period. Mentions include direct tweets and retweets mentioning another user. Any user on Twitter can mention any other user and does not have to be following that user in the social network. This allows us to measure this feature across pairs which do not have a link on any network (na). Twitter users have been shown to exhibit favouritism for a small group of their contacts when it comes to mentions (retweets) [23].
- *Number of common hashtags:* Similarity between users on Twitter can be captured through common interests. Topics are commonly expressed on Twitter with hashtags using the # symbol. We therefore measure the number of instances in which user *i* and user *j* have posted a tweet using the same hashtag. Similar individuals have been shown to have a greater likelihood of having a tie through the principles of homophily [26].
- *Number of colocations:* The number of times two users have checked into the same venue within a given time window. In order to reduce false positives, we consider a shorter time window of 1 hour only. Two users who appear at the same place, at the same time on multiple occasions, have a higher likelihood of knowing each other (and therefore having a link on social media). We weight each colocation on the popularity of a place in terms of the total user visits, to reduce the probability that colocation is by chance at a large hub venue such as an airport or train station. The importance of colocations has been highlighted in discovering social ties as well as place-focused communities [27].
- *Distance:* Human mobility and distance play an important role in the formation of links, both online and offline, and have been shown to be highly indicative of social ties and informative for link prediction [28]. We calculate the distance between the geographic coordinates of two users’ most frequent check-in locations as the Haversine distance, the most common measure of great-circle spherical distance: $\textit{dist}_{ij} = \textit{haversine} (\textit{lat}_{i},\textit{lon}_{i}, \textit{lat}_{j},\textit{lon}_{j})$, where the coordinate pairs for $i,j$ are those of the places where users with more than two check-ins have checked in most frequently, equivalent to the mode in the multiset of the venues where they have checked in. This allows us to minimise data loss motivated by the typical long-tail distribution of activities shown in empirical studies of Foursquare [24], while increasing the probability that a most frequent location will emerge, similar to previous related work in the field [29–31].

**Figure 3:**
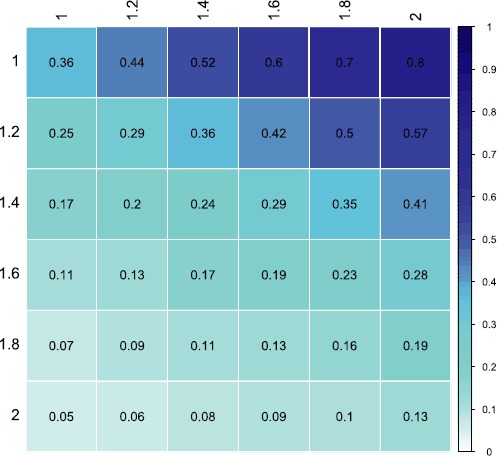
**Exponent matrix for** $\pmb{sim_{GNij}}$ **.** Colour gradient indicates the optimal exponents in terms of difference maximisation between the medians of the multiplex and non-existent link types - $|Md_{tf}-Md_{na}|$.

We additionally consider two geo-social features, which merge information from the Twitter social network and the Foursquare location network. In order to capture the tie strength between a pair of users in the multilayer network, we consider their similarity based on the social layer, or the *number of common hashtags*, denoted by ${\textit{sim}}_{ij}$ and their spatial similarity, or the *distance* between their most frequented venues on Foursquare, denoted by $\textit{dist}_{ij}$. We draw inspiration from gravity models in transportation studies where the attraction between two entities is proportional to the importance of their interaction over their distance [32]. In a similar manner, we aim to identify such an attraction force in the formation of links. Firstly, we define the global similarity as the Twitter similarity over Foursquare distance as:

5 $$ \textit{sim}_{GNij} = \frac{\textit{sim}_{ij}^{a}}{\textit{dist}_{ij}^{b}}, $$

where exponents $a,b$ are chosen based on the context of at hand. In our case, *a* is the potential for the similarity measure to reflect a reciprocal link between two users, whereas *b* is a parameter related to how well connected the two venues are and therefore how significant the distance between them is, similar to the gravity model’s original use in transportation [32]. In the present work, we set the exponents $a=2, b=1$ after optimising for the exponents that maximise the difference between the median values of multiplex links (tf) and no link (na). Figure 3 shows how these results vary across different exponents *a* and *b* in the range $[1,2]$.

We additionally construct a feature which captures the complete interaction across layers of social networks:

6 $$ \textit{int}_{GNij} = \sum_{\alpha}^{M} k^{\alpha}|\textit{int}^{\alpha}_{ij}|, $$

where *int* can be any type of interaction between *i* and *j* in layer *α* and interactions are summed across layers and weighted by a constant *k* for each layer. This allows for adjustments based on the weighted importance of an interaction, specific to the context of the measurement. In our case we consider mentions and colocations as the interactions across layers and a coefficient $k=1$ for both layers as we would like to maintain the empirical properties of interactions and after optimising for a number of different coefficients.

**Figure 4:**
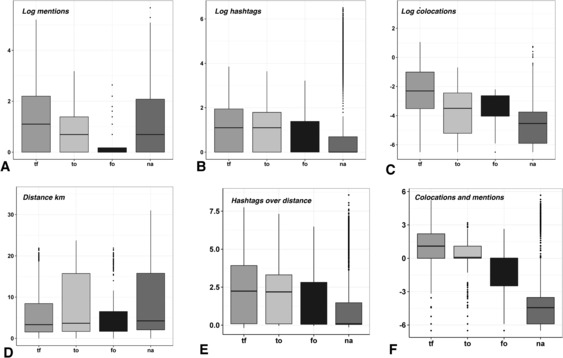
**Interaction features’ distribution for each link type.** Panel Figure A-C show the distributions of Twitter mentions (A) Common hashtags (B), and Number of colocations (C) in log scale. Panel Figure D shows the distribution of distance in km between the home locations of users according to the type of link they have (top 10% of distances are excluded for figure readability), and Figure E and F show the distribution of the multilayer similarity and interaction features.

In Figure 4, we observe the four types of spatial and social interaction on the two social networking services as well as the two geo-social features in the order in which they were presented. Each box-and-whiskers plot represents an interaction between multiplex links (*tf*), Twitter only (*to*), Foursquare only (*fo*), and unconnected pairs (*na*) on the x axis. On the y axis we can observe the distribution divided in four quartiles, representing 25% of values each. The dark line in the middle of the box represents the median of the distribution, while the dots are the outliers, where the definition for an outlier is a value which is less than the first quartile or greater than the third quartile by more than 1.5 times the interquartile range between quartile 3 and 1. The ‘whiskers’ represent the top and bottom quartiles, while the boxes are the middle quartiles of the distribution.

In terms of Twitter mentions (Figure 4(A)), multiplex ties (tf) exhibit higher values of mentions than any other group, including the Twitter only group (to) with a median value of 10^1^ and top-quartile values above 10^4^. Pairs of users connected only on Foursquare (fo) do not typically mention each other on Twitter although this is made possible by the service. On the other hand, mentions are just as common between users who are not connected on any network (na) as between those who are connected on both(tf), which may be as a result of mentioning celebrities and other commercial accounts. This, however, is not the case for *hashtags* (Figure 4(B)), where we find that almost all of unconnected users share 10 hashtags or less with the exception of outliers. While *mentions* are more discriminative between multiplex links (tf) and single-layer connectivity (to and fo), *hashtags* are better at distinguishing between links and non-links (na) in terms of median values.

With regard to Foursquare spatial interaction in Figure 4(C) and (D), multiplex ties (tf) have the highest probability of multiple *colocations* with a median value of 10^−3.8^. Despite being weighted by the popularity of a venue, values in the top quartile of unconnected pairs (na) are relatively high with respect to other link types. However, in terms of median values there is still a distinction between the different levels of multiplexity which each link type represents. On the other hand, while *distance* (Figure 4(D)) does not vary much in terms of median values for the different link types, based on the top quartiles of the distributions across link types, it appears that Foursquare only pairs (fo) are more likely to frequent locations close to each other, closely followed by multiplex link pairs (tf) where distances for both are below 20 km. Twitter only (to) and unconnected pairs frequent locations similarly further away. This indicates that both Foursquare spatial features are better at distinguishing multiplex links and native Foursquare links than other link types based on the distributions observed.

In Figure 4(E) and (F) we can compare the geo-social features we defined above to the single-layer social and geographic features observed. Firstly, we observe the distribution of the $\textit{sim}_{GNij}$ measure integrating similarity and distance as factors of attraction between pairs of users. We can distinguish between link types mainly based on the maximum value in the top quartile of the distributions in Figure 4(E), where we observe that the maximum values for multiplex links are higher than any other link type (over 7.5), whereas the maximum value for unconnected pairs is approximately 4 while the median is 0. This shows that only values with low similarity and high distance fall below 0, whereas most pairs of users have less negligible similarity where values around 1 indicate a balance between distance and similarity.

In Figure 4(F) the distinction between different link types in the distributions of values is more striking than for any of the single-layer features. We can see that each median value is significantly different - multiplex links (tf) are the highest with a median of 10^1.5^, followed by to links (10^0^), fo links (10^−1.5^) and finally non-present links (10^−4^). This satisfies two desirable properties for link prediction - distinct thresholds between link types, and a discriminative threshold between the non-existent links (na) and all other link types, on which to base binary decisions of the presence/absence of a link.

## Multilayer approach to link prediction

The problem of link prediction in online social networks has been actively researched in the past decade, following its ignition by the seminal work of Liben-Nowell and Kleinberg [6]. Since then, has been applied to various platforms and services. For instance, in [9] the authors exploit place features in location-based services to recommend friendships and in similar spirit the authors in [33] show how using both location and social information from the same network significantly improves link prediction, while in [34] a new model based on supervised random walks is proposed to predict new links in Facebook. Link prediction has also been approached in the multidimensional setting [12] and in multi-relational networks [13], however, these works build on features that are endogenous to the system that hosts the network of users.

Drawing upon these works, we train and test on heterogeneous and fundamentally different network layers from two distinct platforms - social network Twitter and location-based social network Foursquare - by mining features from both. Our approach differs in that it frames the link prediction task across layers in the context of multilayer networks, rather than partitions of the same network. Having empirically shown the value of the different features in distinguishing between different link types above, here we approach the question of how this information can be used to predict links across layers of social networks. We evaluate the likelihood of forming a social tie as a process that depends on a union of factors, using the Foursquare, Twitter, and multilayer features we have defined up until now in a supervised learning approach, and comparing their predictive power in each feature set respectively.

### Prediction space

The main motivation for considering multiple social networks in a multilayer construct is that each layer carries with it additional heterogeneous information about the links between the same users, which can potentially enhance the predictive model. In the context of our work we have two distinct layers of information - the spatial movements of users from Foursquare and their parallel social interactions on Twitter. We are interested in exploring whether by using spatial features from one network layer (Foursquare), we are able to predict links on the social network layer (Twitter), and vice versa. In light of the multilayer nature of OSNs, we are also interested in whether we can achieve better prediction by combining features from multiple networks.

Formally, for two users in the multilayer network $i,j \in\mathcal{M}$, where $V^{\mathcal{M}}$ are the nodes (users) that are present in any layer of the multilayer network, we employ a set of features in a supervised learning framework that output a score $r_{ij}^{\alpha}$ so that all possible pairs of users $V^{\mathcal{M}} \times V^{\mathcal{M}}$ are ranked according to their expectation of having a link $e_{ij}^{\alpha}$ on a specific layer *α* in the network. We specify and evaluate two distinct prediction tasks:

(1) We rank pairs of users based on their interaction on one network layer in order to predict a link on the other. This entails (a) training on spatial mobility interactions to predict social links on Twitter, and (b) training on social interaction features on Twitter to test on Foursquare links.

(2) We rank pairs of users based on their interaction on both network layers in order to predict a link across both (a multiplex link). We train on three sets of features - spatial interactions, social interactions, and multilayer features which are summarised in Table 2.

**Table 2:** **Summary of link features. We denote the Twitter neighbourhood as** $\pmb{\Gamma^{T}}$ **and the Foursquare neighbourhood as** $\pmb{\Gamma^{F}}$

**Twitter features**	
*mentions*	$|\textit{mentions}_{ij}|$
*hashtags*	$|\textit{hashtags}_{ij}|$
*overlap*	$\frac{|\Gamma_{i}^{T} \cap \Gamma_{j}^{T}|}{|\Gamma_{i}^{T} \cup \Gamma_{j}^{T}|}$
*aa*_*sim*	$\sum_{z \in \Gamma_{i}^{T} \cap \Gamma_{j}^{T}} \frac{1}{\log(|\Gamma_{z}^{T}|)}$

**Foursquare features**	
*colocs*	$|\textit{colocations}_{ij}|$
*dist*	$\textit{haversine}(\textit{lat}_{i},\textit{lon}_{i}, \textit{lat}_{j}, \textit{lon}_{j})$
*overlap*	$\frac{|\Gamma_{i}^{F} \cap \Gamma_{j}^{F}|}{|\Gamma_{i}^{F} \cup \Gamma_{j}^{F}|}$
*aa*_*sim*	$\sum_{z \in \Gamma_{i}^{F} \cap \Gamma_{j}^{F}} \frac{1}{\log(|\Gamma_{z}^{F}|)}$

**Multilayer features**	
$\textit{int}_{GNij} $	$\sum_{\alpha}^{M} |int^{\alpha}_{ij}|$
${\textit{sim}}_{GNij}$	$\frac{\textit{sim}_{ij}^{a}}{\textit{dist}_{ij}^{b}}$
*overlap*	$\frac{|\Gamma_{CNi} \cap \Gamma_{CNj}|}{|\Gamma_{CNi} \cup \Gamma_{CNj}|}$
*aa*_*sim*	$\sum_{z \in \Gamma_{CNi} \cap \Gamma_{CNj}} \frac{1}{\log(|\Gamma_{CNz}|)}$

We perform our evaluation on the three datasets described in Table 1 for the cities of San Francisco, Chicago, and New York to show performance on these tasks across urban geographies. In terms of algorithmic implementation, we have used public versions of the algorithms available in [35]. Supervised learning methodologies have been proposed as a better alternative to unsupervised models for link prediction [36]. We fit our data to a Random Forest classifier [37], which uses a sub-sampling and averaging technique across a number of tree estimators to improve the predictive accuracy and control over-fitting. Subsampling takes place with replacement and is equal to the training set size. We have optimised across two parameters in each prediction task: the number of tree estimators and the max depth allowed for each estimator.

We additionally use 10-fold stratified cross-validation testing strategy: for each test we train on 90% of the data and test on the remaining 10% and each fold set contains approximately the same percentage of samples of each target class as the complete set since the number of prediction items in the data are in the order of $|V^{\mathcal{M}}|^{2}$. For every test case, the user pairs are ranked according to the scores returned by the classifier for the positive class label (i.e., for an existing link), and subsequently, all possible thresholds of probability in terms of true positive (TP) and false positive (FP) values rate are plotted against each other as Receiver Operating Characteristic (ROC) curves. We use Area Under the Curve (AUC) scores from these curves to report the relative performance of each task by averaging the results across all folds, where we are interested in the fraction of positive examples correctly classified as opposed to the fraction of negative examples incorrectly classified. ROC analysis can provide insight about how well the classifier can be expected to perform in general, at a variety of different class imbalance ratios and therefore, against different random baselines that could correspond to these ratios.

### Multilayer link prediction

**Figure 5:**
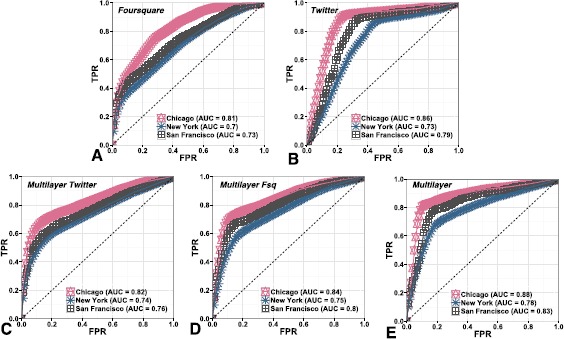
**Link prediction results.** ROC curves for the Random Forest classifier and Area Under the Curve (AUC) scores for each city dataset. Panel Figure A shows the results for predicting Foursquare links using Twitter features, while panel Figure B displays the results for the reverse task of predicting Twitter links using the Foursquare geographical features. Figures C-E focus on the second prediction task - predicting multiplex links using Twitter features (C), using Foursquare features (D) and using multilayer features (E).

We present our evaluation using ROC curves and the corresponding Area Under the Curve (AUC) scores across cities, shown in Figure 5. First, we train on the Twitter social interaction features summarised in Table 2 and test on the Foursquare target labels. Formally, for a pair of users *i* and *j* we define a feature vector $\mathbf{x_{ij}}^{\boldsymbol {\alpha}}$ encoding the values of the users’ feature scores on layer *α* in the multilayer network. We also specify a target label $y_{ij}^{\beta}\in\{-1,+1\}$ representing whether the user pair is connected on the *β* layer under prediction. We use the supervised Random Forest classifier (best performance achieved with 45 tree estimators, allowing for a maximum tree depth = 25 each) to predict links from one layer using features from the other.

Figure 5(A) shows the ROC curves and respective AUC scores for each city in predicting Foursquare links from Twitter features, ranging between 0.7 for the New York dataset to 0.81 for Chicago, and 0.73 for San Francisco. These results represent the probability that the classifier will rank higher a randomly chosen positive instance than a randomly chosen negative instance [38]. On the other hand, we consider the reverse task of predicting Twitter links using Foursquare features in Figure 5(B), where we obtain AUC scores of 0.86, 0.73, and 0.79 for the three cities respectively. We observe slightly higher results for Twitter links, and we note that this may be a result of the higher number of Twitter links in our dataset or as a result of the greater difficulty of the inverse task. We compare these results to the traditional single-layer prediction task of Twitter links from Twitter features and Foursquare links from Foursquare features internal to the platform where we achieve an $\mathrm{AUC}= 0.86$ and $\mathrm{AUC}= 0.88$ on average between cities with the same Random Forest set-up. This shows that our performance across services is comparable to that within the service itself.

We have observed in our preceding analysis on link types that those pairs connected only on Foursquare do not exhibit strong interaction on Twitter by exchanging a low number of mentions and having low neighbourhood overlap, however, those pairs of users connected on both platforms, exhibit high interaction across. We can therefore expect that we have identified a large number of stronger multiplex ties in this task. In our second prediction task, we test this assumption by observing if we are able to achieve higher predictive power across cities when testing on the presence or absence of a multiplex link. Formally, given a feature vector $\mathbf{x_{ij}}$, we would like to predict a target label $y_{ij} \in\{-1,+1\}$, where a link exists on both layers (+1) or not (−1). In Figure 5(C) and (D) we can observe that we are able to achieve greater predictive power using Twitter features in predicting multiplex links than Foursquare links in Figure 5(A) and in using Foursquare features in Figure 5(B), with the highest AUC scores of 0.82 and 0.84 for each set respectively. We also note that the Foursquare spatial features perform slightly better than the social interaction features for Twitter, which places importance on the discriminative power of spatial interactions as also observed in the first part of our analysis. This confirms our assumption that multiplex links are easier to identify than single layer links by using the same algorithmic set-up and shows that the strength of multiplex ties exhibited in the first part of our analysis can be used to predict links across networks.

Finally, we can see that using multilayer and geo-social features which employ both spatial and social interactions from the two heterogeneous platforms can outperform both single layer sets in predicting multiplex links (highest $\mathrm{AUC} = 0.88$ for Chicago). It is intuitive that when using information from both layers the prediction of multiplex links becomes easier and it is often the case that such multilayer network data is not available. However, we have also shown that we can achieve relatively good results using only social or only geographic information.

In order to evaluate the information added by our proposed features as compared to the previously widely used Adamic-Adar and overlap metrics, we compare our prediction results thus far with a simplified model using the Adamic-Adar and overlap features alone, while using the same predictive framework, and compare the change in average AUC scores between cities. For our first prediction task of using the Twitter social layer features to predict links on the spatial Foursquare layer, we achieve an AUC score of 0.68 when using $aa\_{\textit{sim}}$ and $overlap$ features alone as compared to $\mathrm{AUC}=0.8$ when using the full feature set including interactions. For our second task of using the Foursquare spatial features to predict links on the Twitter social layer, we obtain an AUC score of 0.65 when using the two structural features alone as opposed to $\mathrm{AUC}=0.75$ on average across cities when using the full model. This indicates that our additional interaction features add significantly to the predictive power of the model.

When predicting the presence of a multilayer link between pairs of users, using the structural Adamic-Adar and overlap features alone, we achieve an AUC of 0.7 for the social Twitter layer, 0.71 for the spatial Foursquare layer, and 0.69 for our multilayer configuration. When compared to our full feature model ($\mathrm{AUC}=0.77,0.8, \mbox{and } 0.83$ respectively), we note a significant improvement in terms of predictive power. In conclusion, the information added by our multilayer interaction features results in a significant improvement over the existing methods based on popular structural features alone.

## Discussion & conclusions

Recently, social media has been increasingly alluded to as an *ecosystem*. The allusion comes from the emergence of multiple OSNs, interacting as a system, while competing for the same resources - users and their attention. We have addressed this system aspect by modelling multiple social networks as a multilayer online social network in this work. Most new OSNs joining the ‘ecosystem’ use contact list integration with external existing networks, such as copying friendships from Facebook through the open graph protocol.^3^ Copying links from pre-existing social networks to new ones results in higher social interaction between copied links than between links created natively in the platform [39]. We propose that augmenting this copied network with a rank of relevance of contacts using multiplexity can provide even further benefits for newly launched services.

In addition to fostering multiplexity, however, new OSNs and especially interest-driven ones such as Pinterest for example, may benefit from similarity-based friend recommendations. In this work, we apply mobility features and neighbourhood similarity from Foursquare to predict links on Twitter and vice versa, highlighting the relationship between similar users across heterogeneous platforms. Similarly in [11], the authors infer types of relationships across different domains such as mobile and co-author networks. Although using a transfer knowledge framework, and not exogenous interaction features like we do, the authors also agree that integrating social theory in the prediction framework can greatly improve results.

The strength of ties manifested through multiplexity is expressed through a greater intensity of interactions and greater similarity across attributes both the offline [4, 5], and in the online context as we have seen in this work. We have explored a number of features, which take into consideration the multilayer neighbourhood of users in OSNs. The Adamic/Adar coefficient of neighbourhood similarity in its core neighbourhood version proved to be a strong indicator of multiplex ties. Additionally, we introduced combined features, such as the global interaction and similarity over distance, which reflect more distinctively the type of link, which exists between two users, than its single-layer counterparts. These features can be applied across multiple networks and can be flexible in their construction according to the context of the OSNs under consideration.

Media multiplexity is fascinating from the social networks perspective as it can reveal the strength and nature of a social tie given the full communication profile of people across all media they use [4]. Unfortunately, full online and offline communication profiles of individuals were not available and our analysis is limited to two social networks. Nevertheless, we have observed some evidence of media multiplexity manifested in the greater intensity and structural overlap of multiplex links and have gained insight into how we can utilise these properties for link prediction. Certainly, considering more OSNs and further relating media multiplexity to its offline manifestation is one of our future goals, and we believe that with the further integration of social media services and availability of data this will be possible in the near future.

## Footnotes

1
https://foursquare.com/about

2
https://about.twitter.com/company

3
https://developers.facebook.com/docs/opengraph
